# Interference of pseudorabies virus infection on functions of porcine granulosa cells via apoptosis modulated by MAPK signaling pathways

**DOI:** 10.1186/s12985-024-02289-y

**Published:** 2024-01-23

**Authors:** Lingcong Deng, Wenpeng Min, Songyangnian Guo, Jiping Deng, Xiaosong Wu, Dewen Tong, Anwen Yuan, Qing Yang

**Affiliations:** 1 College of Veterinary Medicine, Hunan Agricultural University, 410128 Changsha, Hunan China; 2 College of Veterinary Medicine, Northwest A&F University, 712100 Yangling, Shaanxi China; 3 Research Center of Reverse Vaccinology, College of Veterinary Medicine, Hunan Agricultural University, 410128 Changsha, Hunan China

**Keywords:** Pseudorabies virus, Ovarian granulosa cells, Steroidogenesis, Apoptosis, MAPK signaling pathway

## Abstract

**Background:**

Pseudorabies virus (PRV) is one of the major viral pathogens leading to reproductive disorders in swine. However, little is known about the effects of PRV infection on porcine reproductive system. Ovarian granulosa cells are somatic cells surrounding oocytes in ovary and required for folliculogenesis. The present study aimed to investigate the interference of PRV on functions of porcine ovarian granulosa cells in vitro.

**Methods:**

Primary granulosa cells were isolated from porcine ovaries. To investigate the PRV infectivity, transmission electron microscopy (TEM) was used to check the presence of viral particles, and the expression of viral *gE* gene was detected by quantitative real-time PCR (qPCR) in PRV-inoculated cells. After PRV infection, cell viability was detected by MTS assay, Ki67 for proliferative status was determined by immunofluorescence assay (IFA), cell cycle and apoptosis were detected by flow cytometry, and progesterone (P_4_) and estradiol (E_2_) were determined by radioimmunoassay. The checkpoint genes of cell cycle and apoptosis-related proteins were studied by qPCR and western blotting.

**Results:**

Virus particles were observed in the nucleus and cytoplasm of PRV-infected granulosa cells by TEM imaging, and the expression of viral *gE* gene increased in a time-dependent manner post infection. PRV infection inhibited cell viability and blocked cell cycle at S phase in porcine granulosa cells, accompanied by decreases in expression of Ki67 protein and checkpoint genes related to S phase. Radioimmunoassay revealed decreased levels in P_4_ and E_2_, and the expressions of key steroidogenic enzymes were also down-regulated post PRV-infection. In addition, PRV induced apoptosis with an increase in Bax expression and activation of caspase 9, and the phosphorylation of JNK, ERK and p38 MAPKs were significantly up-regulated in porcine ovarian granulosa cells post PRV infection.

**Conclusions:**

The data indicate that PRV causes infection on porcine ovarian granulosa cells and interferes the cell functions through apoptosis, and the MAPK signaling pathway is involved in the viral pathogenesis.

**Supplementary Information:**

The online version contains supplementary material available at 10.1186/s12985-024-02289-y.

## Background

Pseudorabies virus (PRV) is a member of the family Herpesviridae, subfamily Alphaherpesvirinae, genus Varicellovirus [1]. Although PRV has a broad host range and infects most mammals, pigs are the only natural reservoir. PRV infects pigs of all ages, causing diarrhea, respiratory failure, death, and neurological disorder among piglets. Viral envelope glycoproteins play important roles in the processes of entry and egress of herpesviruses [2]. Herpesvirus glycoprotein gE is necessary for virulence and virion replication, involving in syncytia formation, nerve invasion and cell-to-cell spreading [3, 4]. Almost all wildtypes of PRV strains express gE, therefore seroprevalence of gE antibody is often used for differential diagnosis of wildtype PRV infection. Tan et al. collected data from 108 studies and found an average yield of 29.87% positive rate of PRV infection in pigs from 2011 to 2021 [5].

PRV severely disturbs swine reproduction, such as infertility, abortion, and stillbirth, resulting in significant economic losses worldwide [6, 7]. Intrauterine inoculation of PRV in natural breeding gilts causes lesions in the reproductive tract with lymphohistiocytic vaginitis and endometritis, and lymphoplasmacytic aggregates in the corpora lutea of ovary [8]. PRV also causes placental lesions in pregnant sows [9]. In addition, researchers have reported that PRV infects germ cells of testes [10], alveolar macrophages [11, 12], and mononuclear cells from lymphoid tissues [13]. In our previous study, we also found that pseudorabies virus infection was prevalent in breeding pigs. The PRV-gE seropositivity was 28% (1,650/5,921) in sows, 20% (474/2,370) in gilts, and 26% (352/1,354) in boars, respectively [14].

Mammalian follicle is the basic functional unit of ovary and the site of oogenesis. Ovarian granulosa cells are the largest cell population in follicles and synthesize hormones and growth factors, providing a stable microenvironment for oocyte maturation and follicular development [15, 16]. Processes of proliferation and apoptosis naturally occur in ovarian granulosa cells. An imbalance between these processes leads to ovarian pathological changes. As the main reproductive organ, ovary is susceptible to several pathogens. In mice, Zika virus replicated in ovary and infected the granulosa cells as well as the theca cells in antral follicles, which significantly damaged ovarian structure and further disrupted the estrous cycle and prolonged pregnancy by causing disordered ovarian steroidogenesis [17]. Moreover, the expression of porcine circovirus type 2 antigen was detectable in ovarian tissues of naturally infected gilts [18]. However, it is still unknown whether PRV could infect the ovarian granulosa cells and disrupt cell function, leading to abnormal ovarian function in sows. In the present study, we investigated the effects of PRV infection on cell growth, hormone secretion, and apoptosis in porcine ovarian granulosa cells.

## Materials and methods

### Antibodies

The primary antibodies against FSH-R, Ki67, pseudorabies virus, and β-actin were purchased from Abcam (Cambridge, MA, USA). The antibodies against-cleaved caspase 9, caspase 9, and p38 were obtained from Proteintech Group (Chicago, IL, USA); antibodies against JNK and phosphor-JNK (Thr183/Tyr185) were purchased from Cell Signaling Technology, Inc (Boston, MA, USA). The antibody against phosphor-p38 (Y182) and anti-rabbit lgG-HRP second antibody (sc-2305) were purchased from Santa Cruz Biotechnology (Dallas, TX, USA). Cy3 conjugated goat anti-rabbit IgG (H + L) secondary antibody was from Servicebio Technology Co., Ltd (Wuhan, China).

### Isolation of porcine ovarian granulosa cells

Porcine ovaries were collected from a local slaughterhouse under veterinarian control. All experimental procedures were performed with the approval of the Ethical Committee of Animal Experiments, Hunan Agricultural University (No. 43201701). Ovaries were kept in sterile saline containing 2% penicillin-streptomycin (Beyotime Biotechnology, Shanghai, China) at 37 °C and transported to the laboratory within 1 h. Ovarian granulosa cells were isolated and cultured according to the techniques described by others [19] with minor modifications. Briefly, follicular fluid was collected from 3-mm to 5-mm diameter follicles. Cells precipitated naturally in 37 °C water bath for 15 min followed by centrifuge. Cell pellet was resuspended in erythrocyte lysate (Beyotime, Shanghai, China) to remove blood cells. Then, cells were washed with PBS twice, collected, and cultured in DMEM medium supplemented with 10% fetal bovine serum (FBS) (Gibco-Invitrogen, Carlsbad, CA, USA) and 1% penicillin-streptomycin at 37 °C and 5% CO_2_ in a humidified incubator (Thermo Fisher Scientific, MA, USA). The isolated cells were subjected to screening for animal pathogens including PRV, African swine fever virus (ASFV), and porcine circovirus type 2 (PCV2). Cells were also identified using a specific marker follicle-stimulating hormone receptor (FSH-R) by indirect immunofluorescence assay (IFA).

### PRV infectivity by transmission electron microscopy imaging

Pseudorabies virus (PRV-YY strain) was gifted from Dr. Xinglong Yu, which was propagated in PK-15 cells and measured the titer (2 × 10^− 7^/0.1 mL TCID_50_). Virus stock was diluted using DMEM medium. Porcine ovarian granulosa cells were infected with the virus at 5 TCID_50_, 10 TCID_50_, and 50 TCID_50_ as indicated in absence of FBS for 1.5 h. PK-15 cell lysate with the same dilution was used to treat the granulosa cells as the control. The unbound virus was removed. Cells were cultured in DMEM supplemented with 2% FBS at 37 °C for the indicated time points.

The presence of viral particles in granulosa cells following PRV infection was observed using transmission electron microscopy (TCM). Briefly, granulosa cells were collected 24 h post infection with a titer of 50 TCID_50_. Cells were fixed in 2.5% cold glutaraldehyde (G1102, Servicebio, Wuhan, China), and post-fixed in 1% osmium tetroxide solution (18456, Ted Pella Inc, CA, USA). The pellets were embedded in SPI-Pon™ 812 Epoxy Resin Monomer (SPI, PA, USA) after washes with ultrapure water and dehydration with graded ethanol. The blocks were polymerized at 65 °C for 48 h. Ultrathin (~ 80 nm) sections were cut using an ultramicrotome (Ultracut UC7, Leica, Wetzlar, Germany) and mounted on grids. Sections were stained with 2.5% uranyl acetate and 2.6% lead citrate and imaged using a HT7700 Transmission Electron Microscope (Hitachi, Tokyo, Japan).

### Expression of viral *gE* gene in PRV-infected porcine granulosa cells

Expression of PRV *gE* gene was detected in granulosa cells at the indicated hours post infection (hpi). The growth medium was removed, cells were rinsed with 1× PBS twice and viral DNA was extracted using a TIANamp virus DNA/RNA kit (TIANGEN Biotech, Beijing, China) according to the manufacturer’s instructions. The extracted DNA was used as template for quantitative real-time PCR (qPCR) using Vazyme ChamQ™ SYBR®qPCR Master Mix (Vazyme) according to the manufacturer’s instructions. The cycling conditions were 94 °C for 5 min, followed by 35 cycles of 94 °C for 30 s, 60 °C for 30 s, and 72 °C for 30 s, and a final extension of 72 °C for 10 min. The primer information was listed in Table 1.

**Table 1:** Sequences of the primers used in qPCR

Genes	Accession No.	Forword primers (5’-3’)	Reverse primers (5’-3’)
*PRV-gE*	FJ176390.1	TTGTGGGTGGCGTTTTATCTCCGTC	AAGTTGGCGCCCTCGGACACGTTC
*GAPDH*	NM_001206359.1	ACAGGGTGGTGGACCTCATG	GGGTCTGGGATGGAAACTGG
*CDK1*	NC_010456.5	GGGTCAGCTCGCTACTCAAC	AGTGCCCAAAGCTCTGAAAA
*CDK2*	NC_010447.5	GCTTCAGGGGCTAGCTTTTT	AGCCCAGAAGGATTTCAGGT
*CCNE1*	NC_010448.4	AAGTGGCACTGATGTCTCTGTT	CCAAGGCTGATTGCCACACT
*CCNA1*	NC_010453.5	GACAGGGTGTGTGTGTCAGG	GAACGTAGCAAGGGCTTCTG
*CCNB1*	NC_010458.4	TGGTGCACTTTCCTCCTTCT	TTGTAAGCCCTCGATTCACC
*CYP11A1*	NC_010449.5	GTCCCATTTACAGGGAGAAGCTCG	GGCTCCTGACTTCTTCAGCAGG
*3β-HSD*	NC_010446.5	ATCGTCCACTTGTTGCTGGA	TGCTCTGGAGCTTAGAAAATTCC
*StAR*	NC_010457.5	GGAGAGCCGCCAGGAGAATC	CTTCTGCAGGATCTTGATCTTCTTG
*CYP19A1*	NC_010443.5	TGAAGTTGTGCCTTTTGCCAG	AGGACCTGGTATTGAAGATGTGTTT
*17β-HSD*	NC_010447.5	CCAAACAAACATCGCAGGCA	TAGTCCTGGCCGTAGTCCTC

### MTS assay

Cell viability was determined by MTS assay. Briefly, the isolated ovarian granulosa cells were seeded in 96-well plates (1.5 × 10^4^ cells per well). When reached 80% of confluence, cells were inoculated with PRV as indicated for 1.5 h, then washed with PBS and cultured with maintenance medium for 24 h, 36 h, and 48 h. After treatment, 20 µL MTS reagent (Promega Corporation, Madison, WI, USA) was added to each well and cells were incubated at 37 °C for 2 h. The absorbance was read at 490 nm using a microplate reader (Thermo Fisher Scientific, USA).

### Immunofluorescence assay (IFA)

The isolated and PRV-infected cells were fixed with 4% paraformaldehyde for 20 min, and punched with 0.2% Triton X-100 in PBS for 10 min at 37°C. Then, cells were blocked with 3% bovine serum albumin followed by incubation with primary antibodies (FSH-R antibody, 1:200; Ki67 antibody; 1:200). Blocking buffer was substituted for the primary antibodies as a negative control. After PBS wash, ovarian granulosa cells were incubated with the fluorescein-conjugated secondary antibody (goat anti-rabbit secondary antibody, 1:500) for 1 h at room temperature. Finally, samples were washed with PBS, and stained with 4’, 6-diamidino-2-phenylindole (DAPI). Signal was observed and imaged using a fluorescence microscope (Olympus, Japan).

### Flow cytometry

For cell cycle assay, the PRV-infected or Mock-treated porcine ovarian granulosa cells were dissociated with trypsin and fixed in 70% cold ethanol at 4 °C overnight. Then, cell pellet was re-suspended in 500 µL of the propidium iodide (PI)/RNase staining buffer after PBS wash and incubated in the dark at 37 °C for 30 min. The fluorescence of the PI was measured by a FACSCalibur flow cytometer (Becton Dickinson, San-Jose, CA, USA). Percentage of the cell population in each cell cycle phase (G0/G1, S, and G2/M) was calculated from DNA content histograms using FlowJo software (version 10, Tree Star, Ashland, OR, USA). A minimum of 20,000 cells were analyzed.

For apoptosis analysis, cells were harvested after treatment. Cell pellet was resuspended in 400 µL of 1 × binding buffer and double stained with 5 µL FITC-Annexin V (Annexin V-FITC) and 5 µL PI (TransGen Biotech, Beijing, China) in the dark for 20 min at room temperature, and analyzed by flow cytometry. The data were analyzed with the FlowJo software.

### RNA extraction, cDNA synthesis and quantitative real-time PCR

Total cellular RNA was extracted using TransZol Up Plus RNA kits (TransGen Biotech, Beijing, China), and the concentration was measured by a NanoDrop 2000 spectrophotometer (Thermo Fisher Scientific). cDNA template was generated from 500 ng RNA using the HiScript®II Q RT SuperMix for qPCR (+ gDNA wiper) (Vazyme Biotech, Nanjing, China). Quantitative real-time PCR (qPCR) was performed on an ABI Step One Real-Time PCR System (Applied Biosystems, Foster City, CA, USA) using the ChamQTM SYBR® qPCR Master Mix (Vazyme). The PCR cycling conditions were 40 cycles of 95 °C 10 s, 60 °C 30 s, and 72 °C 60 s. The sequences of specific primers are listed in Table 1. The expression of the target genes was normalized to the porcine GAPDH and calculated using the 2^−∆∆Ct^ method.

### Western blot analysis

The PRV-infected or Mock-treated porcine granulosa cells were lysed with a RIPA buffer (Solarbio Science & Technology Co., Ltd., Beijing, China) supplemented with protease inhibitor cocktail (Roche, Basel, Switzerland). Protein concentrations were measured by using a BCA kit (Thermo Fisher, MA, USA). Equal amounts of protein (30 µg) were electrophoresed and separated by SDS-PAGE. Proteins were transferred onto PVDF membranes (Millipore Corp., MA, USA). After blocking with 5% non-fat milk in tris-buffered saline solution containing 0.1% tween-20 (TBST) at room temperature for 1 h, the membranes were incubated with the indicated primary antibodies diluted in blocking buffer (cleaved-caspase 9, caspase 9, Bax, Bcl-2, p-p38, p38, p-JNK, JNK, p-ERK and ERK antibodies, 1:2000; β-actin antibody, 1:5000) overnight at 4 °C. Subsequently, membranes were washed with TBST three times and incubated with the corresponding secondary antibody (1:5000 dilution) at room temperature for 1 h. Protein bands were developed using the UltraSignal Electrochemiluminescent (ECL) Substrate (4 A Biotech Co., Ltd, Beijing, China) and checked by ChemiDoc™ XRS+ (Bio-Rad, USA) system. Levels of proteins were quantified by using Image J software with β-actin as internal control.

### Radioimmunoassay

Cell culture medium was collected after treatment. Levels of progesterone (P_4_) and estradiol (E_2_) in culture medium were measured by radioimmunoassay. The assay was performed by Beijing North Institute of Biological Technology (Beijing, China). The sensitivity and inter- and intra-assay coefficients of variations of E_2_ and P_4_ were as follows: E_2_, 5 pg/ml, < 15%, and < 10%; P_4_, 0.2 ng/ml, < 15%, and < 10%. Each sample was measured in triplicate.

### Statistical analysis

Data are represented as means ± SEM from three independent experiments, and analyzed using Student’s t-test and one-way ANOVA. Statistical analysis was performed using SPSS software (Version 17.0; SPSS, Chicago, IL, USA) and GraphPad Prism 6 (GraphPad Software Inc., San Diego, CA, USA). *P* value under 0.05 was considered statistically significant.

## Result

### PRV infectivity in porcine ovarian granulosa cells

The immunofluorescence staining showed that the isolated cells expressed FSH-R with red fluorescence signals in the cytoplasm and nucleus, no positive staining signal was observed in the control cells (Fig. S1), which indicated that the cells were ovarian granulosa cells and could be used for the subsequent experiments. The isolated cells were also subjected to screening for common porcine pathogens, and cells detected negative for PCV2, ASFV, and PRV were used for further experiments (Fig. S2).

**Fig. 1:**
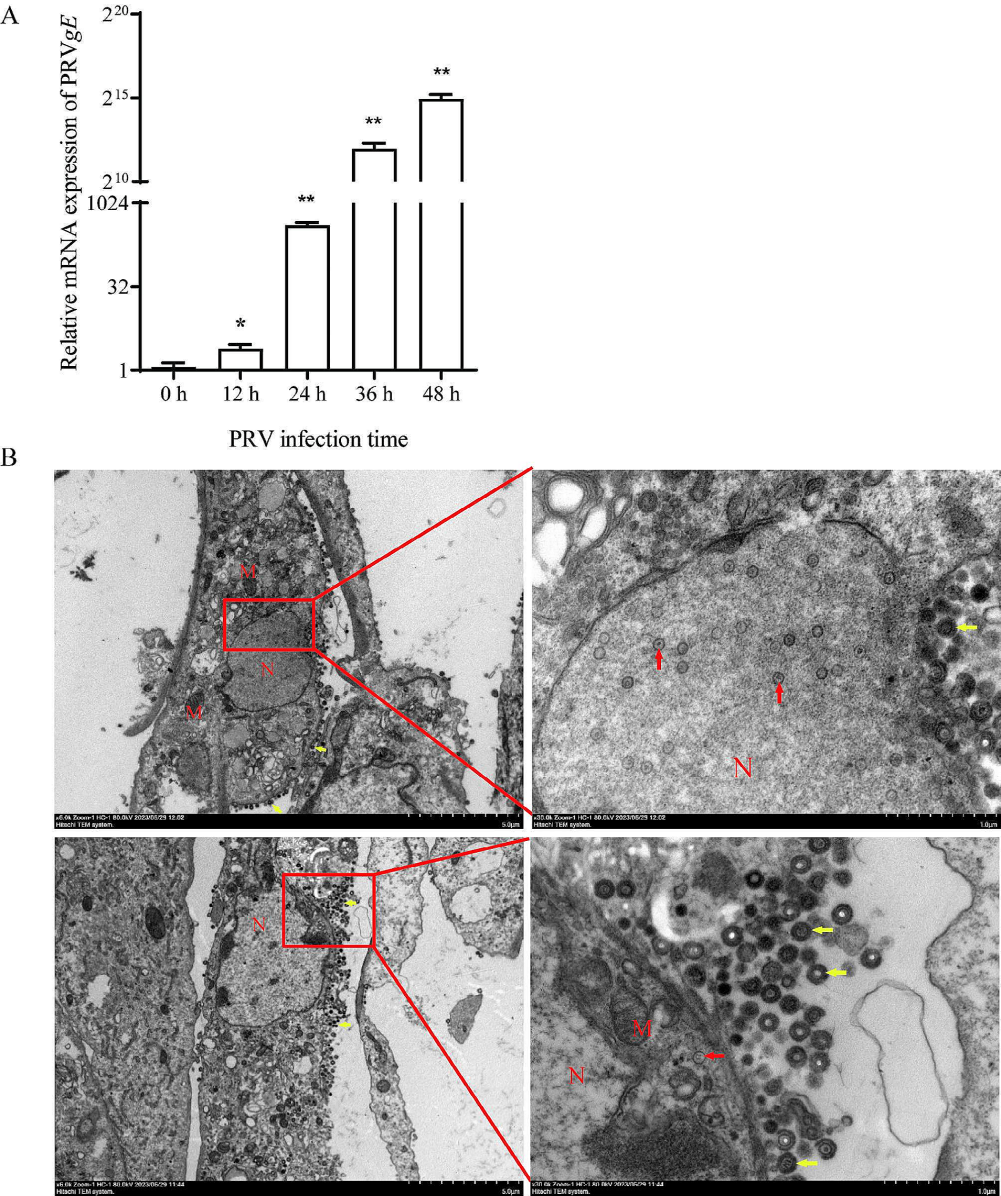
PRV infectivity in porcine ovarian granulosa cells. Granulosa cells were infected with PRV suspension (a titer of 50 TCID_50_) at 37 °C for 1.5 h to allow viral attachment, then collected for viral nucleic acid analysis or for transmission electron microscopy analysis. **(A)** Nucleic acid level of PRV *gE* in porcine granulosa cells infected with PRV at different time points. Statistical analysis was compared with the 0 h group; data represented as mean ± SEM (*n* = 3); * *P* < 0.05, **P < 0.01. **(B)** Transmission electron microscopy (TEM) analysis. Porcine ovarian granulosa cells inoculated with PRV (50 TCID_50_) was observed at 24 h post infection. A large amount of mature viral particles (dark color, about 200 nm in size) were observed, adhering to nuclear membrane of the PRV-infected cell, and immature viral particles presented in the cytoplasm; scale bars were 500 nm (left panel) and 100 nm (right panel), respectively. Yellow arrows indicate mature viral particle in dark color, about 200 nm in size, and red arrows indicate immature viral particle in light color, about 100 nm in size. N: nucleus; M: mitochondria

To investigate the replication of PRV in porcine granulosa cells, real-time quantitative PCR was used to detect the expression of the viral *gE* gene in PRV-infected cells. The results showed that a tiny amount of PRV *gE* could be detected in cells at 0 hpi (as 1.5 h after inoculation) and 12 hpi. At 24 hpi, a high level of viral nucleic acid was detected, which exhibited a time-dependent increase at 36 hpi and 48 hpi (Fig. 1A). TEM imaging of PRV-infected cells showed large numbers of round particles in the cytoplasm; under a high magnification, the particles showed typical morphology of the mature PRV virus with elliptically shaped, surrounded by icosahedral capsid containing the viral genome, ~ 200 nm in size with dark color; immature viral particles were observed in the nucleus with light color, ~ 100 nm in size (Fig. 1B).

### PRV inhibited cell proliferation of porcine ovarian granulosa cells

**Fig. 2:**
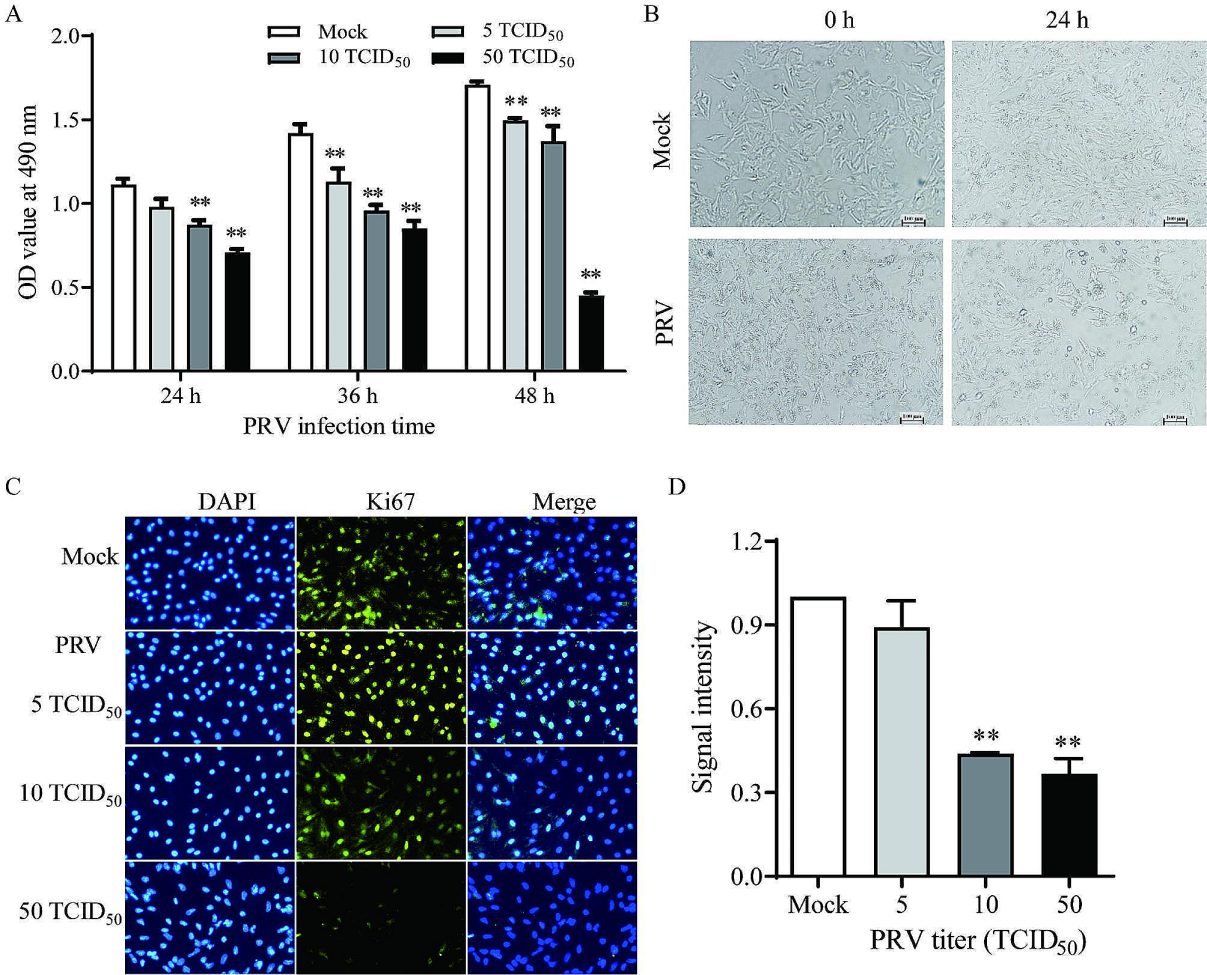
PRV infection inhibited cell viability and Ki67 level in porcine ovarian granulosa cells. Porcine granulosa cells were infected with PRV suspension (5, 10 and 50 TCID_50_) for the indicated time points. **(A)** Cell viability was detected by MTS assay. Data represented as mean ± SEM (*n* = 3); Asterisks indicate a significant difference compared to the Mock group. **P < 0.01. **(B)** PRV infection (50 TCID_50_) caused obvious cytopathic effects in granulosa cells at 24 h post infection. **(C)** Expression of Ki67 in granulosa cells following PRV infection at 36 h by IFA. (**D)** Quantification of the fluorescence signal by Image J. Data show as mean ± SEM (*n* = 3); * *P* < 0.05, ** *P* < 0.01

Cell viability of the PRV-infected ovarian granulosa cells was significantly decreased at the indicated time points when compared to the matched Mock-treated group (*P* < 0.01) (Fig. 2A). The results indicated that PRV infection caused a decrease in cell viability of the porcine ovarian granulosa cells in a dose- and time-dependent manner. Typical cytopathic effects (CPE) were observed in granulosa cells at 24 hpi with a titer of 50 TCID_50_ of PRV (Fig. 2B).

Ki67 is commonly used as a cellular proliferation indicator. The expression of Ki67 was also detected in the PRV-infected porcine ovarian granulosa cells by IFA. The results showed that the fluorescence signal of Ki67 decreased significantly when the granulosa cells were infected with 10 or 50 TCID_50_ PRV at 36 hpi (*P* < 0.01), there was no significant difference when cells infected with 5 TCID_50_ PRV (*P* ˃ 0.05) (Fig. 2C and D). No fluorescence signal was detected in the control cells without Ki67 antibody incubation (data not shown).

### PRV induced S phase cell cycle arrest in porcine ovarian granulosa cells

**Fig. 3:**
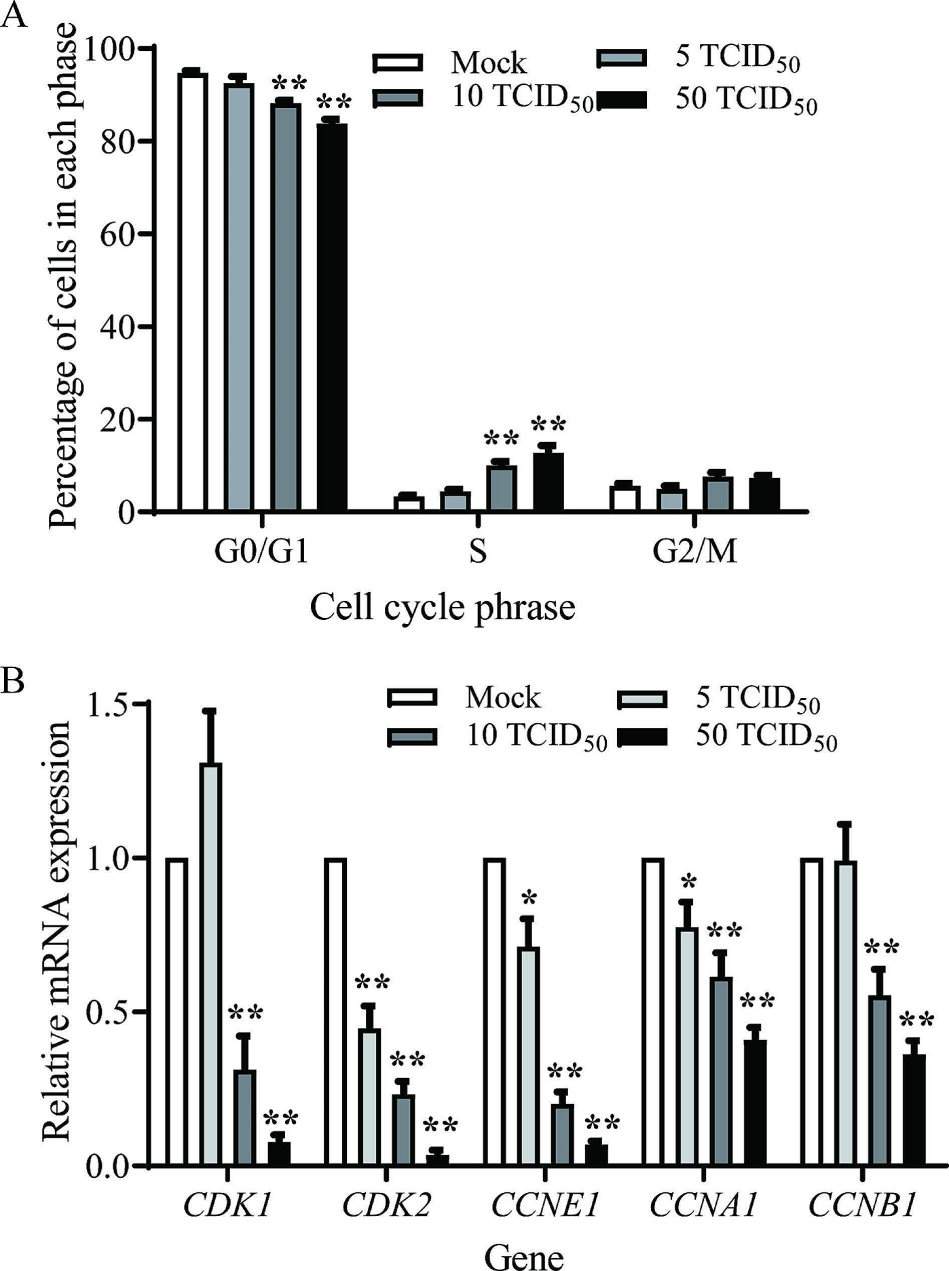
PRV induced S phase cell cycle arrest in porcine granulosa cells. **(A)** Proportions of porcine granulosa cells in each phrase were analyzed by flow cytometry. **(B)** S phase-related genes were detected by RT-qPCR assay after PRV infection. Data were mean ± SEM (*n* = 3). Significant differences between Mock- and PRV-infection are indicated by ** *P* < 0.01

To confirm the inhibition of PRV on porcine ovarian granulosa cells growth, we investigated the effect of PRV on cell cycle. The percentage of porcine granulosa cells at S phage increased significantly when cells were infected with 10 or 50 TCID_50_ PRV at 36 hpi (*P* < 0.01), with a marked reduction in G0/G1 phase (*P* < 0.01) (Fig. 3A). qPCR assay further revealed that PRV infection down-regulated the mRNA expression of several S phase cell cycle-related genes including *CDK1*, *CDK2*, *CCNA1*, *CCNB1*, and *CCNE1* in PRV-infected granulosa cells with the indicated titers (*P* < 0.01) (Fig. 3B).

### PRV impaired steroidogenesis in porcine ovarian granulosa cells

**Fig. 4:**
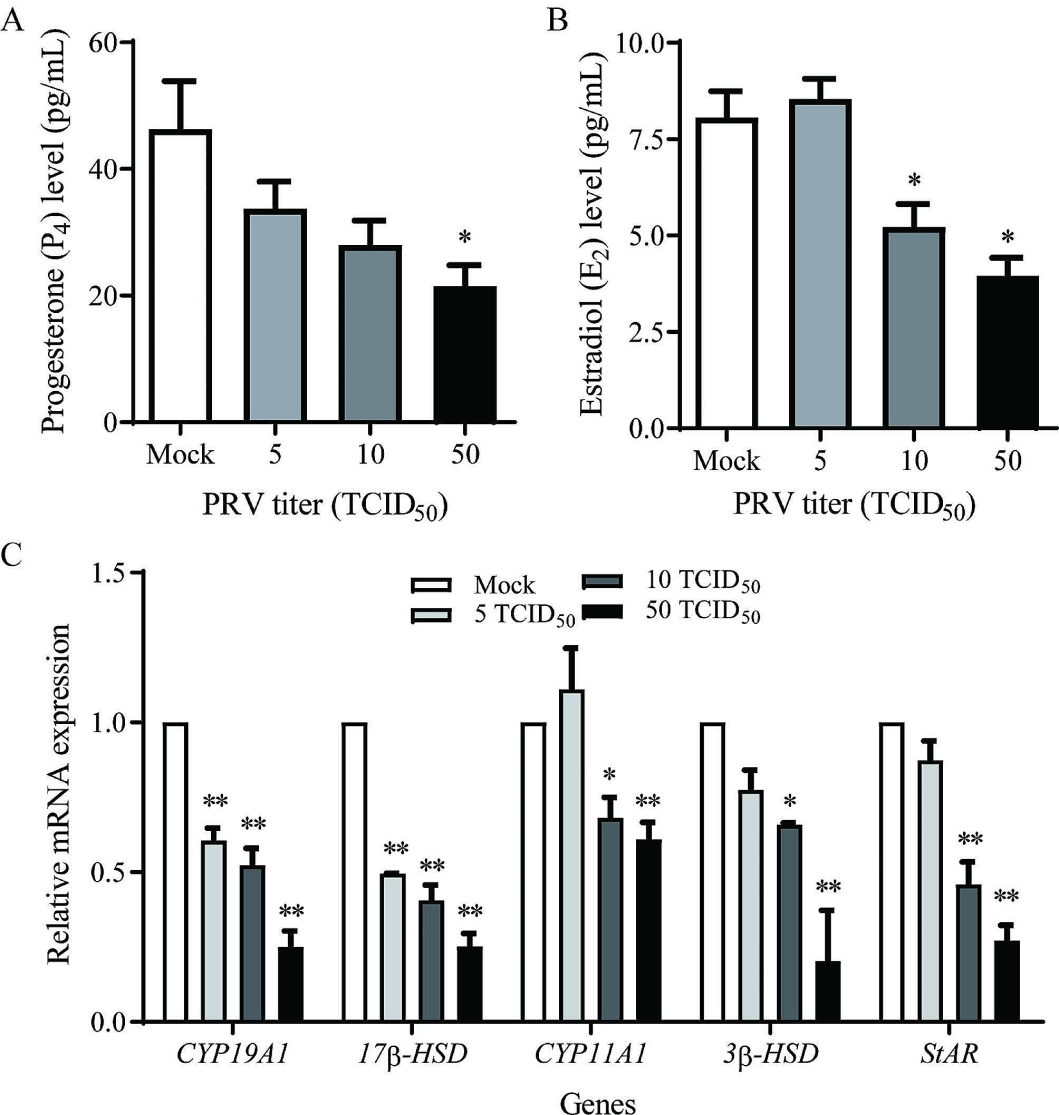
PRV infection impaired steroidogenesis in porcine ovarian granulosa cells. Porcine granulosa cells were infected with PRV at titers 5, 10 and 50 TCID_50_ for 1.5 h and then cultured for 36 h, steroid hormones in the cell culture medium were measured by radioimmunoassay, and the expression of steroidogenesis-related enzymes in cells were detected by qPCR. **(A)** Progesterone (P_4_) level. **(B)** Estradiol (E_2_) level. **(C)** Expression of *CYP19A1*, *17β-HSD*, *CYP11A1*, *3β-HSD* and *StAR* mRNA. Expression of the target genes were normalized to the corresponding *GAPDH* level. Data were mean ± SEM (*n* = 3). Significant differences between Mock- and PRV-infection are indicated by * *P* < 0.05, ** *P* < 0.01

To determine the effect of PRV on steroidogenesis in porcine granulosa cells, we firstly measured the secretion of progesterone and estradiol in the culture medium by radioimmunoassay. As shown in Fig. 4A, there was a decline trend in progesterone secretion when the porcine granulosa cells were infected with increased titers of PRV, which was significantly decreased at the titer of 50 TCID_50_ (*P* < 0.05). The accumulation of estradiol was similar to that of progesterone, which exhibited a significant decline when cells were infected with 10 or 50 TCID_50_ of PRV (*P* < 0.05) (Fig. 4B). We further detected the expressions of steroid hormone synthesis-related genes including *17β-HSD*, *CYP11A1*, *3β-HSD*, *CYP19A1*, and *StAR* by RT-qPCR assay. The results showed that mRNA levels of the five steroidogenesis-related genes were significantly decreased when the porcine granulosa cells were infected with a titer of 10 or 50 TCID_50_ PRV (*P* < 0.01), and *17β-HSD and CYP19A1* mRNAs also decreased in PRV-infected granulosa cells at 5 TCID_50_ (Fig. 4C).

### PRV infection promoted apoptosis of porcine ovarian granulosa cells

**Fig. 5:**
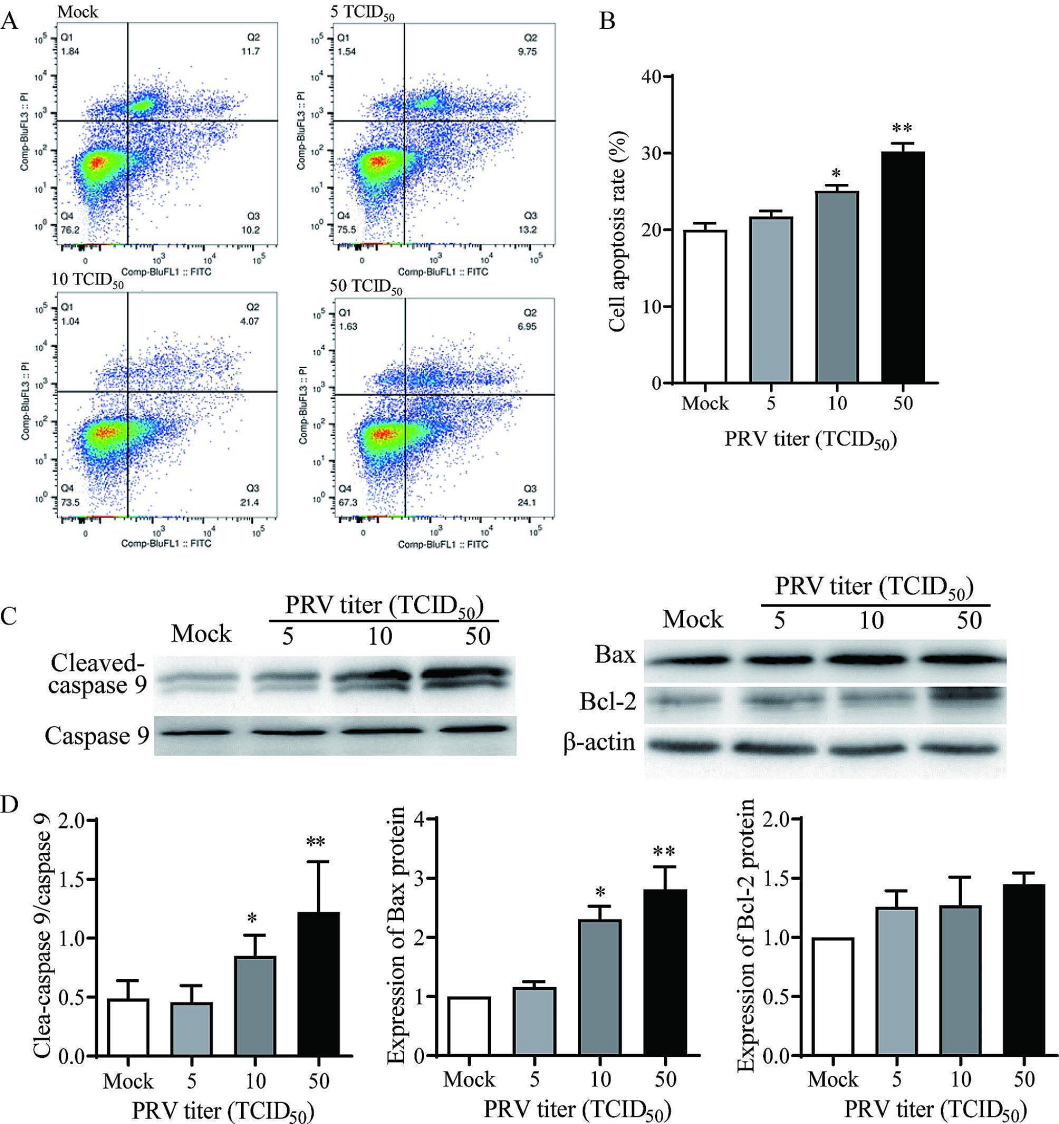
Effect of PRV on apoptosis of porcine granulosa cells. Apoptosis level was measured in porcine granulosa cells infected with PRV at titers 5, 10, 50 TCID_50_ or Mock using flow cytometry. **(A)** Representative image of flow cytometry-based analysis. **(B)** Quantitative analysis of the apoptotic cells stained as Annexin V-FITC positive. **(C** and **D)** Expression of apoptosis-related proteins including cleaved-caspase 9, caspase 9, Bax and Bcl-2 was determined by Western blotting. Data were shown as mean ± SEM values of three independent experiments. Significant differences between Mock- and PRV-infection are indicated by * *P* < 0.05, ** *P* < 0.01

In the present study, we studied the effect of PRV infection on apoptosis in the primary porcine ovarian granulosa cells, and also detected the expressions of the apoptosis-related proteins. The results from flow cytometry showed that the percentage of apoptotic cells increased significantly in PRV-infected cells with titers of 10 (*P* < 0.05) or 50 TCID_50_ (*P* < 0.01) (Fig. 5A and B). The expression of Bax, a pro-apoptotic protein, was increased (*P* < 0.05), and the caspase-9 was activated (*P* < 0.05) (Fig. 5C and D) when the porcine granulosa cells were infected with 10 or 50 TCID_50_ of PRV. There was no significant difference in the anti-apoptotic protein level of Bcl-2 (*P* ˃ 0.05) following virus infection (Fig. 5C and D).

### PRV infection activated the MAPK signaling in porcine ovarian granulosa cells

**Fig. 6:**
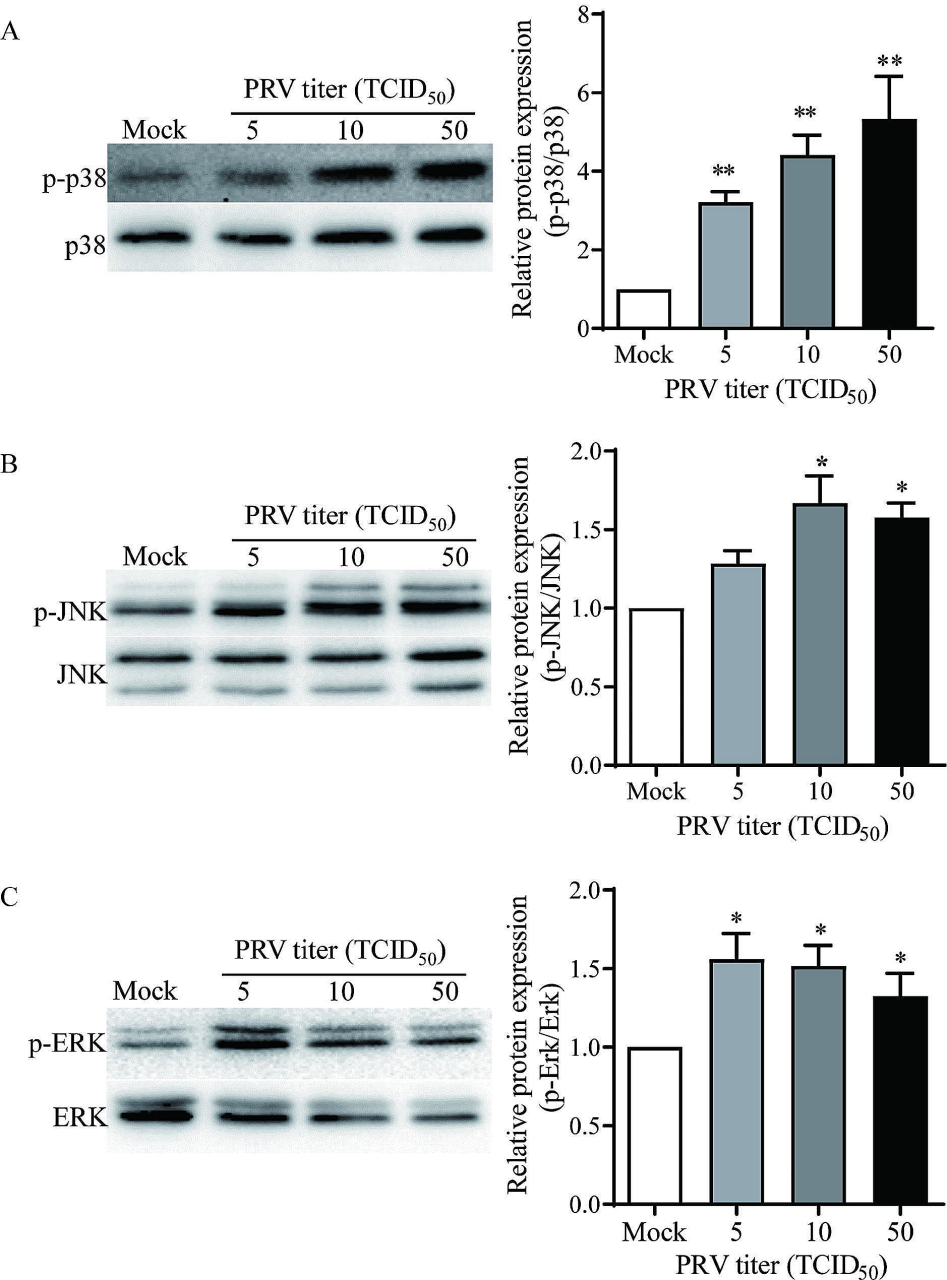
PRV infection activated the MAPK signaling in porcine granulosa cells. The expression of three major MAPKs family members-ERK, p38 and JNK was detected in porcine granulosa cells following PRV infection with PRV at titers 5, 10, 50 TCID_50_ by Western blot analysis. Representative blot images and quantification for p-p38/p38 **(A)**, p-JNK/JNK **(B)** and p-ERK/ERK **(C).** Data were shown as mean ± SEM values of three independent experiments. Significant differences between Mock- and PRV-infection are indicated by * *P* < 0.05, ** *P* < 0.01

A group of viruses interact with MAPK family members including ERK, p38, and JNK to manipulate cellular functions in its favor [20]. In the present study, the phosphorylated levels of ERK, p38, and JNK were significantly promoted in porcine granulosa cells following PRV infection (Fig. 6).

## Discussion

Follicle development is accompanied by a remarkable increase in number of granulosa cells, there are only thousands of granulosa cells in the early antral follicle, whereas at least one hundred thousand granulosa cells in the antral follicle [21]. Steroidogenesis is the main function of granulosa cells. Progesterone, androstenedione, and estradiol are produced in response to the stimulation of the FSH and LH, which can be impaired under the stimulation of exogenous inducers or viral infection [22, 23]. It is well known that PRV suppresses the proliferation of swine testicular and kidney epithelial cells [10, 24], and mouse splenocytes [25]. The present findings also showed infectivity of PRV in porcine ovarian granulosa cells. The expression of the PRV *gE* gene was significantly increased in the granulosa cells following PRV infection at 12 to 48 h, and the mature and un-assembled viral particles were observed in the cells. Moreover, obvious CPE was observed in the granulosa cells after PRV infection. In the PRV-infected granulosa cells, cell proliferation was inhibited and the expression of cell proliferation marker Ki67 was downregulated.

Viruses employ a variety of mechanisms to control or manipulate host cell cycle to maximize their replication in cells [26, 27]. S phase is a critical phase during cell cycle progression which provides a beneficial cellular environment for viral replication [27–29]. The present findings showed that PRV induced cell cycle arrest at S phage in porcine granulosa cells. It needs further investigation whether this is a strategy employed by PRV for viral production. Cell cycle progression is controlled by a series of protein complexes composed of cyclins and cyclin-dependent kinases (CDKs) [30]. The expression of several key regulators of the cell cycle including cyclins (CCNA1, CCNB1, and CCNE1) and CDKs (CDK1/2) were down-regulated in the PRV-infected porcine granulosa cells. These results indicate that S phase is a critical phase to regulate proliferation of the PRV-infected porcine granulosa cells that is closely related to the inhibition of cyclins and CDKs.

Apoptosis is an important process regulating the pathogenesis of virus infection [31, 32]. Several herpesviruses induced cell-specific apoptosis in skin cells, lymphocytes, and bovine kidney epithelial cells through MAPK, cell death, mitochondrial signaling pathways [33–35]. MAPK signaling is intricately regulated by protein phosphorylation. Viruses stimulate MAPKs to regulate a wide range of gene expression in host or virus for viral multiplication [20, 36]. MAPK family, ERK, plays distinct roles in herpesvirus infection in different cells. Activation of ERK is essential for infection of herpes simplex virus type 1 (HSV-1) in neuronal cells and human herpesvirus-8 in human dermal microvascular endothelial cells and fibroblasts [37, 38]. However, HSV-1 suppressed ERK activity in human dermal fibroblasts [39]. Rhesus monkey rhadinovirus (RRV), another herpesvirus, also activates ERK pathway, which is essential for the production of lytic viral proteins and virions. Paradoxically, knockdown of intracellular ERK fails to inhibit RRV production [40]. In the present study, PRV induced apoptosis of the porcine granulosa cells in a dose manner, in which the MAPK signaling was activated in response to viral infection. Meanwhile, levels of the pro-apoptotic protein Bax and the phosphorylated caspase 9 were up-regulated after PRV infection, which was also reported in other herpes viruses [35, 41].

Host actively initiates apoptosis to remove the infected cells for surviving, while viruses may interfere with host genes by inhibiting apoptosis with increase of the progeny virions production and release [42, 43]. PRV infection induces apoptosis in swine cells both in vitro and in vivo [32]. Meanwhile, PRV infection inhibits apoptosis in the trigeminal ganglionic neurons of swine during acute infection in vivo [44]. PRV US3 protein kinase mediated inhibition of apoptosis without affecting the production of infectious virus [45, 46]. The involvement of PRV in precise regulation of apoptosis of the porcine granulosa cells needs to be investigated further. In addition to proliferation, granulosa cells differentiate into appropriate stages during follicular development. Whether PRV interferes the differentiation of granulosa cells also needed to be disclosed.

## Conclusion

We primarily demonstrated that PRV propagates in porcine primary granulosa cells and inhibits the proliferation of porcine ovarian granulosa cells, which is related to S phase arrest in the cell cycle. PRV infection decreases the steroidogenesis in porcine ovarian granulosa cells and downregulates the expression of steroidogenesis-related enzymes. In addition, we also suggest that PRV promotes apoptosis in porcine ovarian granulosa cells and MAPK-mitochondrial apoptosis pathway plays important roles in PRV pathogenesis.

### Electronic supplementary material

Below is the link to the electronic supplementary material.

Supplementary Material 1

Supplementary Material 2

Supplementary Material 3

## Data Availability

The datasets used and/or analyzed during the current study are available from the corresponding author upon reasonable request.

## Abbreviations

PRV: Pseudorabies virus
qPCR: Quantitative real-time PCR
TEM: Transmission electron microscopy
MTS: 3-(4,5-dimethylthiazol-2-yl)-5-(3-carboxymethoxyphenyl)-2-(4-sulfophenyl)-2 H-tetrazolium
JNK: C-Jun N-terminal kinase
MAPK: Microtubule-associated protein kinase
FSH-R: Follicle-stimulating hormone receptor
RIPA: Radioimmunoprecipitation assay buffer
BCA: Bicinchoninic acid assay
CDK1: Cyclin-dependent kinases 1
CDK2: Cyclin-dependent kinases 2
CCNA1: Cyclin A1
CCNB1: Cyclin B1
CCNE1: Cyclin E1
17β-HSD: 17β-hydroxysteroid dehydrogenases
CYP11A1: Cytochrome P450 11A1
3β-HSD: 3β-hydroxysteroid dehydrogenases
CYP19A1: Cytochrome P450 19A1
Bcl-2: B-cell lymphoma-2
