# Supplementary figures and images for: Interference of pseudorabies virus infection on functions of porcine granulosa cells via apoptosis modulated by MAPK signaling pathways
Source: Virol J. 2024 Jan 23;21:25. doi: 10.1186/s12985-024-02289-y (PMC10807058; doi:10.1186/s12985-024-02289-y)

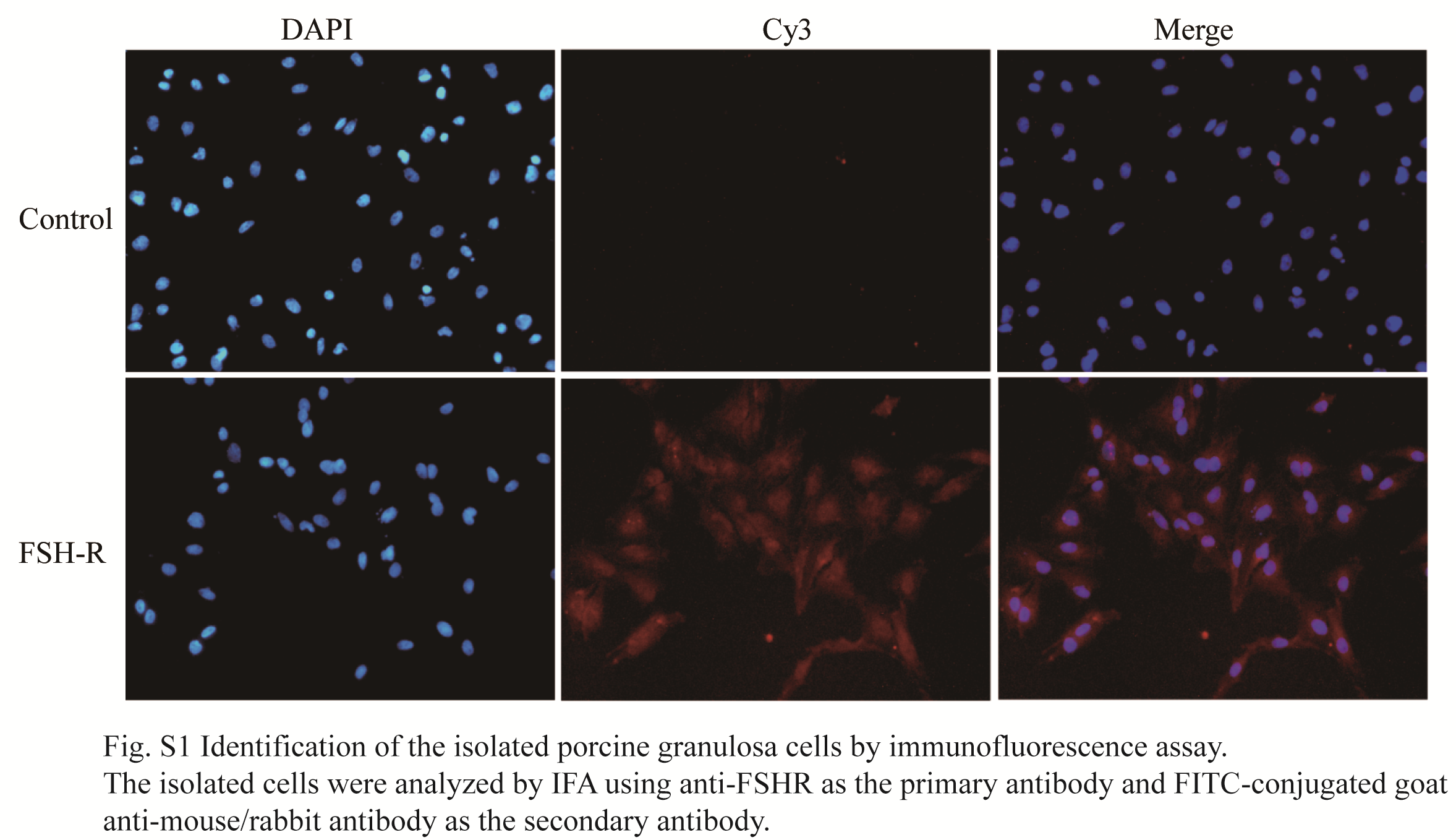

Supplement: Supplementary file 1 — Supplementary Material 1 [file 12985_2024_2289_MOESM1_ESM.png]

Figure 5 Raw image

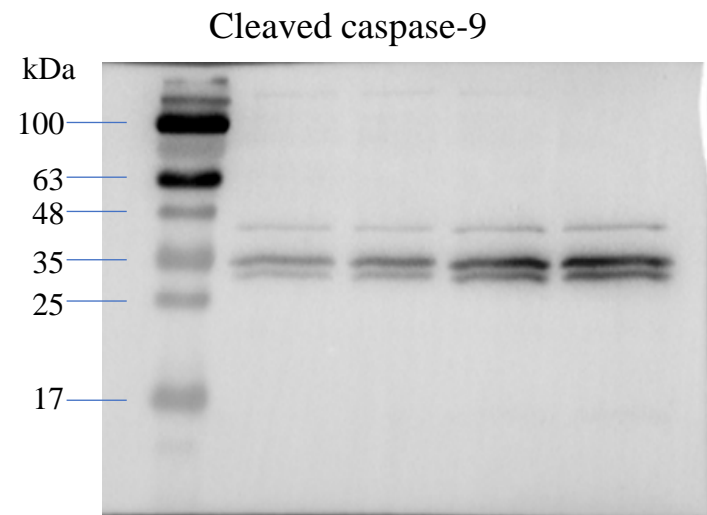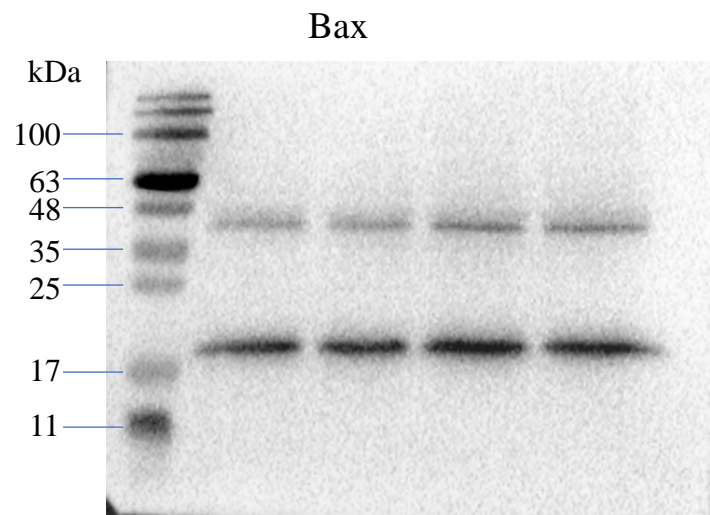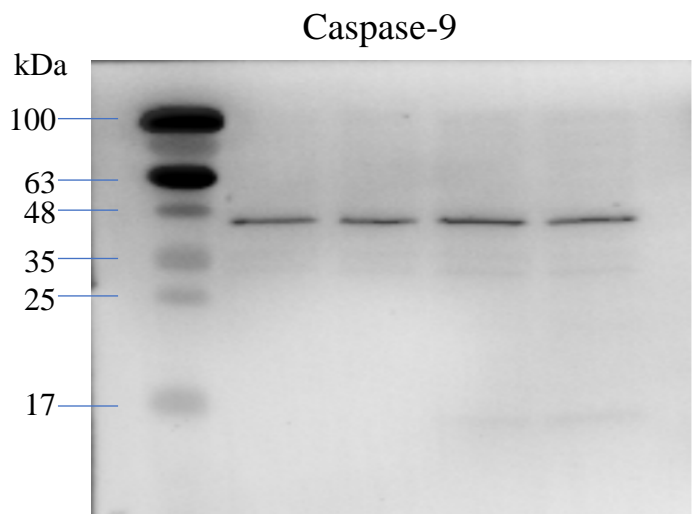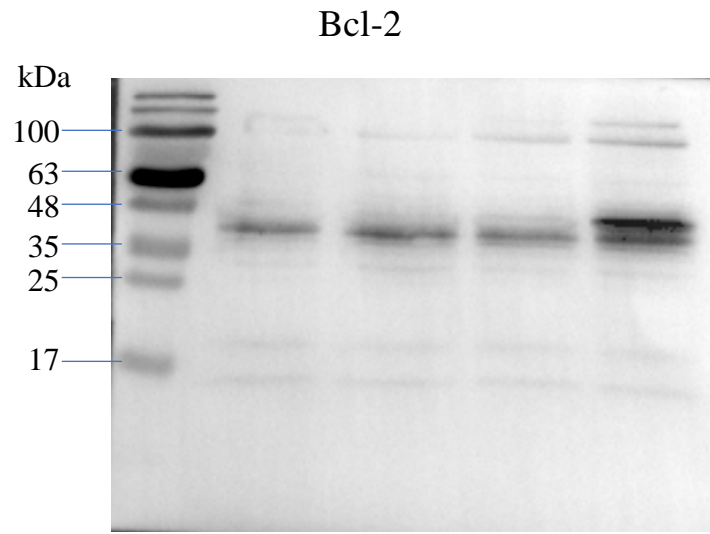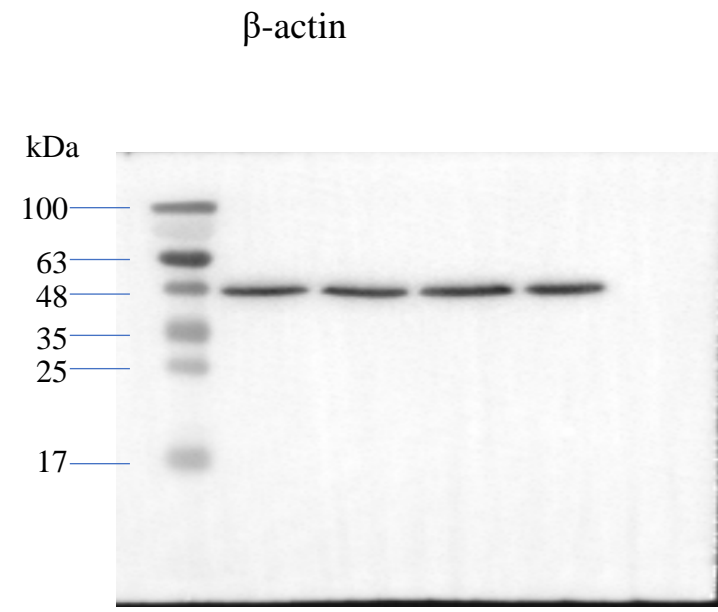

Figure 6 Raw image

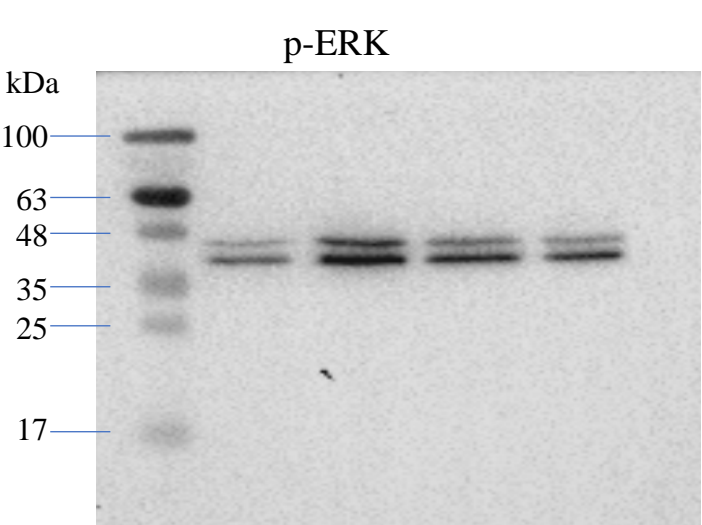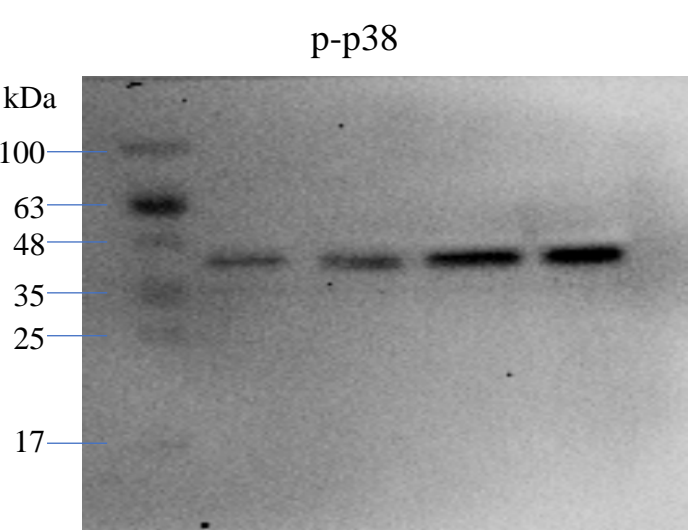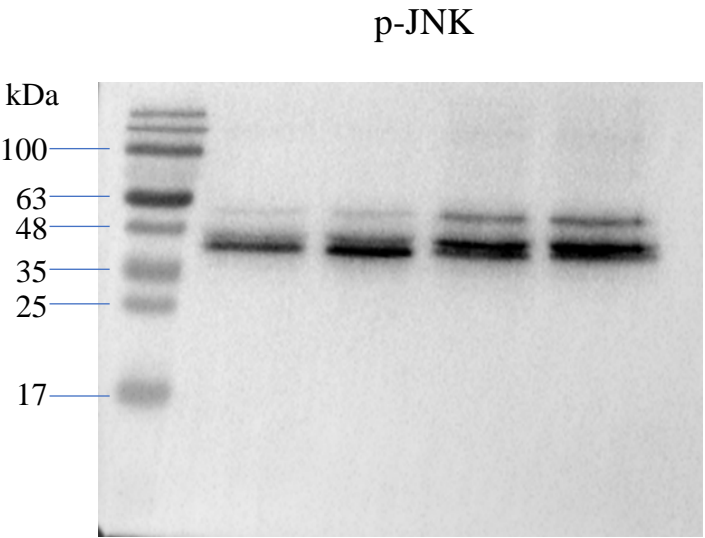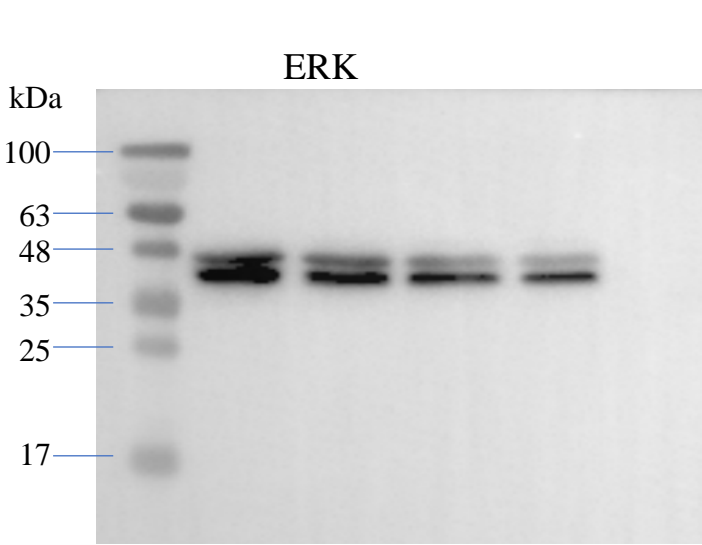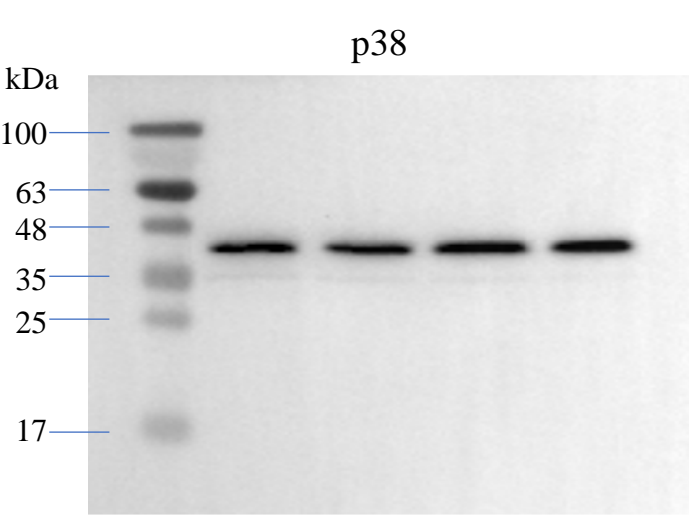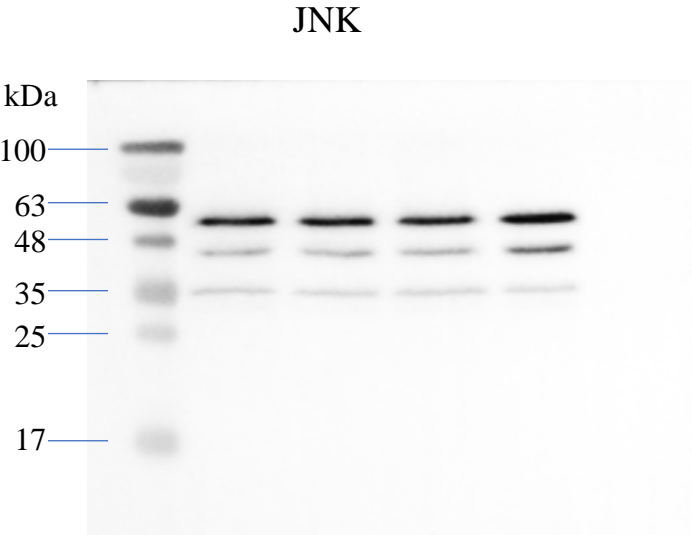

Supplement: Supplementary file 2 — Supplementary Material 2 [file 12985_2024_2289_MOESM2_ESM.pdf]

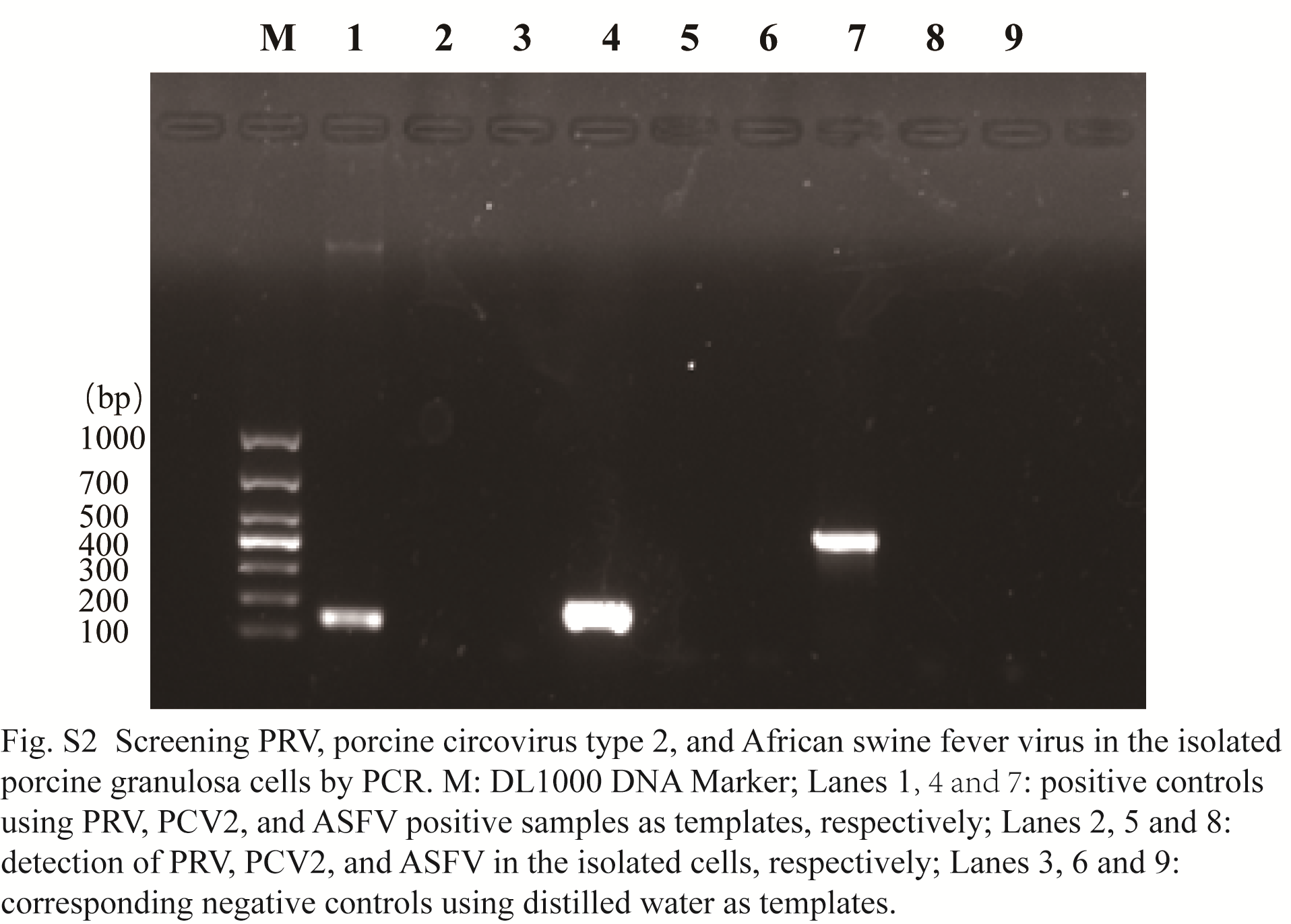

Supplement: Supplementary file 3 — Supplementary Material 3 [file 12985_2024_2289_MOESM3_ESM.png]
